# Supplementary material for: Structural basis for negative regulation of the Escherichia coli maltose system
Source: Nat Commun. 2023 Aug 15;14:4925. doi: 10.1038/s41467-023-40447-y (PMC10427625; doi:10.1038/s41467-023-40447-y)
Supplement: Supplementary file 1 — Supplementary Information [file 41467_2023_40447_MOESM1_ESM.pdf]

Supplementary Information

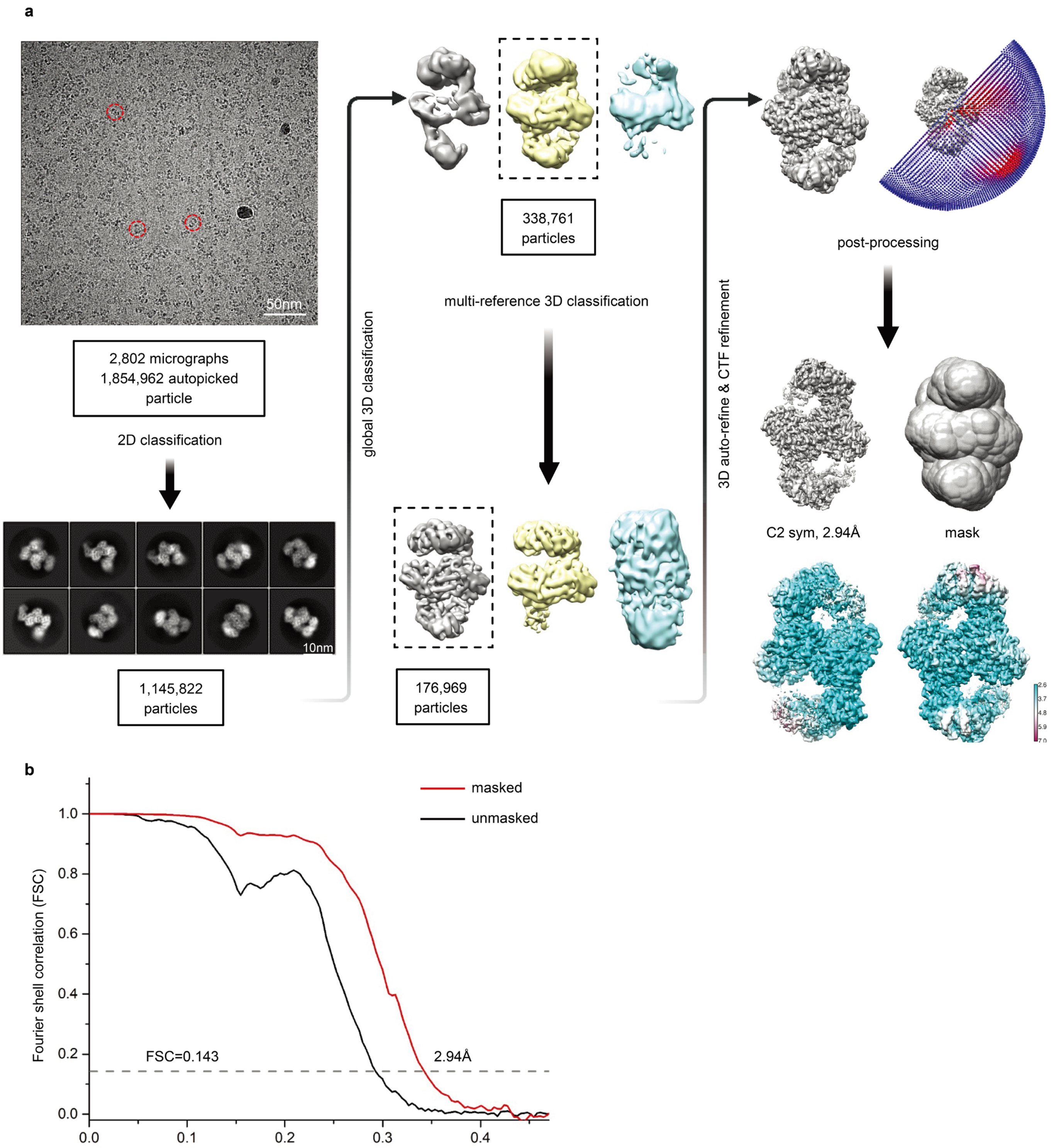

Supplementary Fig. 1 Cryo-EM workflow for the MalT-MalY complex. a, Flowchart for cryo-EM data processing and 3D reconstruction of the MalT-MalY complex. **b**, FSC curve at 0.143 of the 3D reconstructions of the MalT-MalY complex.

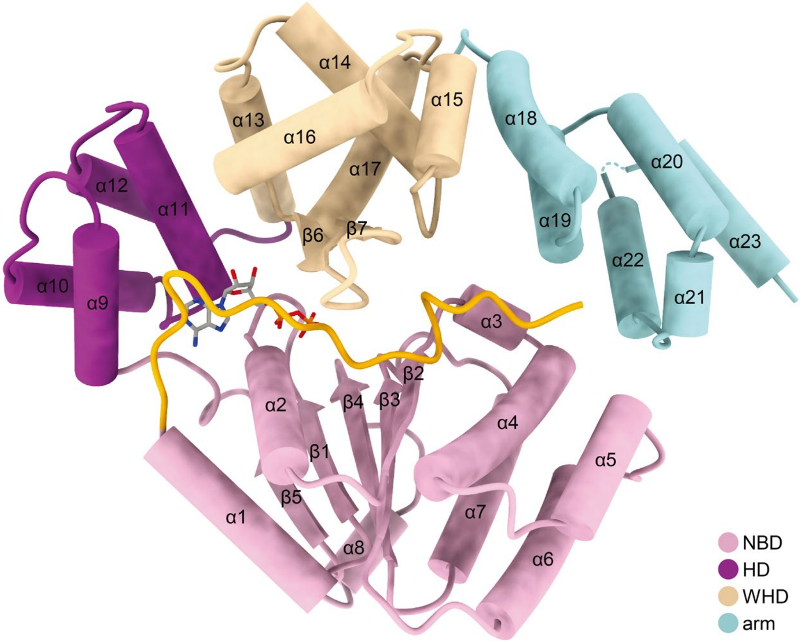

Supplementary Fig. 2 MalT with secondary structural elements labelled. Numbering of α-helices and β-strands is based on their order (from the N- to the C-terminal end) in the structure. Colors of each protein domain are indicated, the N-terminal segment of NBD is highlighted in yellow.

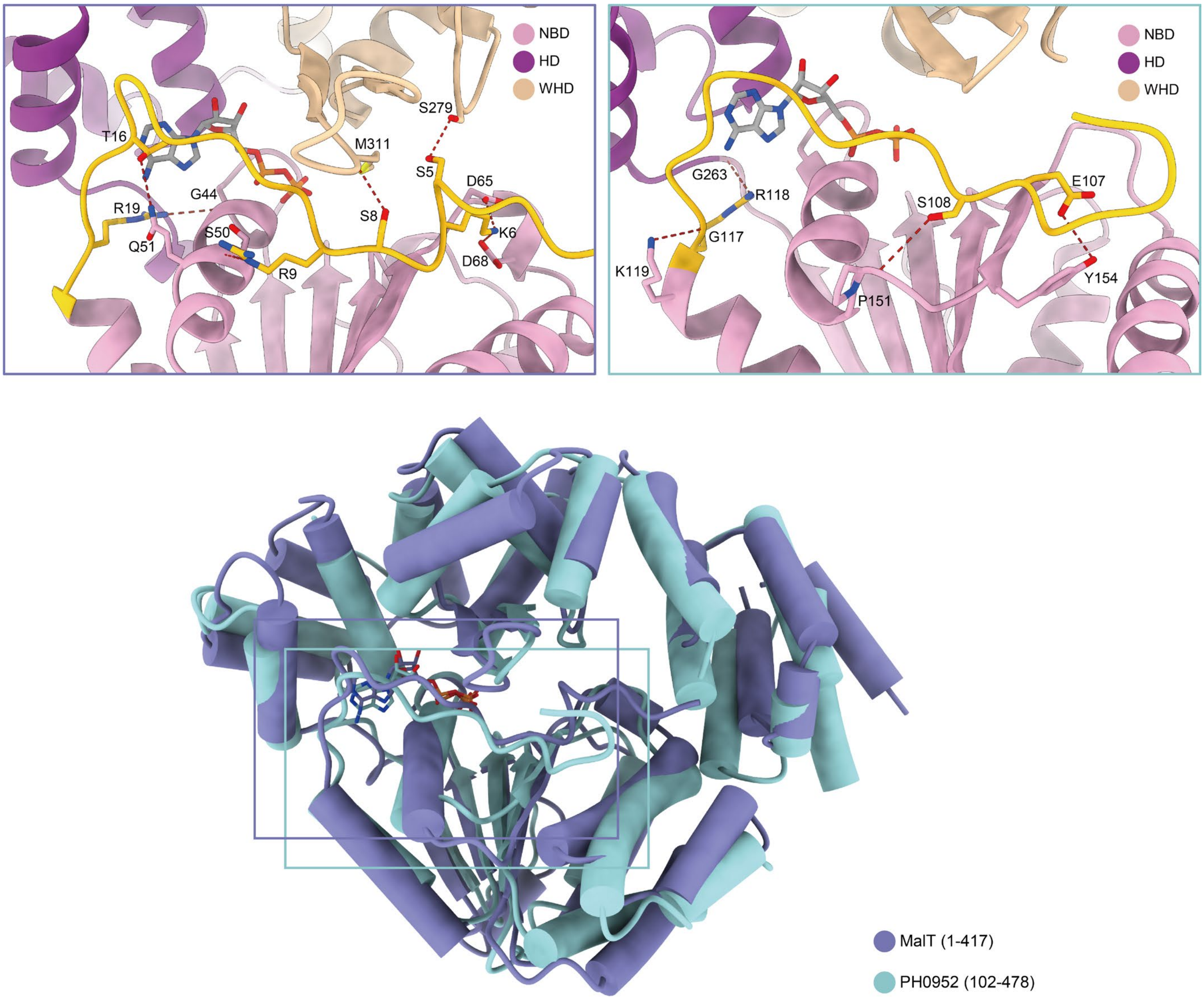

Supplementary Fig. 3 Structural alignment of MalT with inactive PH0952 (PDB: 6MFV). MalT and PH0952 are colored in cyan and slate, respectively (lower panel). Polar interactions formed between the N-terminal segment (highlighted in yellow) and other domains of MalT (upper panel, left) or PH0952 (upper panel, right) are represented by red dashed lines. Color of each protein domain is indicated.

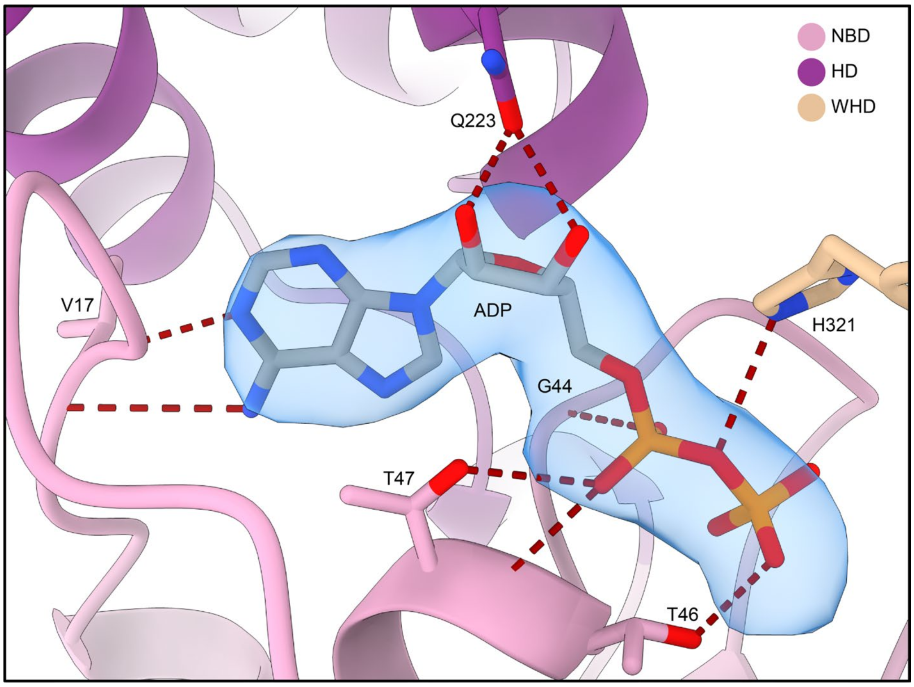

Supplementary Fig. 4 Recognition of ADP by MalT. Detailed interactions of ADP with residues from MalT NBD, HD, and WHD. Polar interactions are represented by red dashed lines. The density of ADP is shown in transparent surface view.

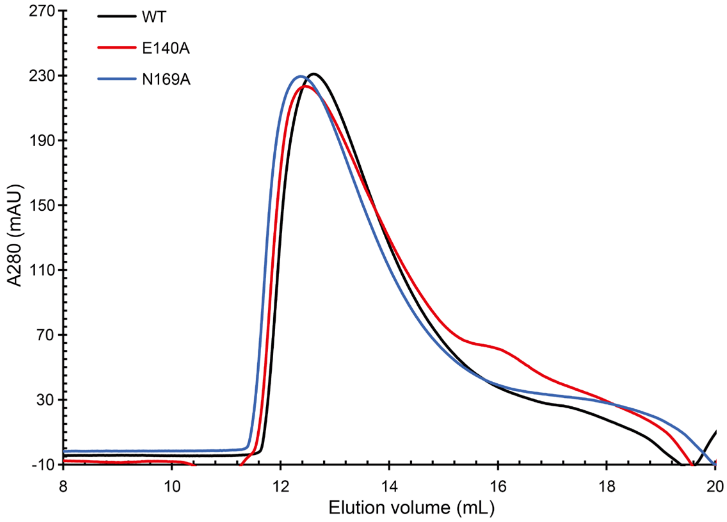

Supplementary Fig. 5 Gel filtration analyses of MalT WT, E140A, and N169A. The same molar amount of MalT WT, E140A, and N169A proteins were pre-incubated and subjected to gel filtration analyses using a Superose 6 Increase 10/300 GL column in the presence of 1 mM maltotriose and 0.4 mM ATP.

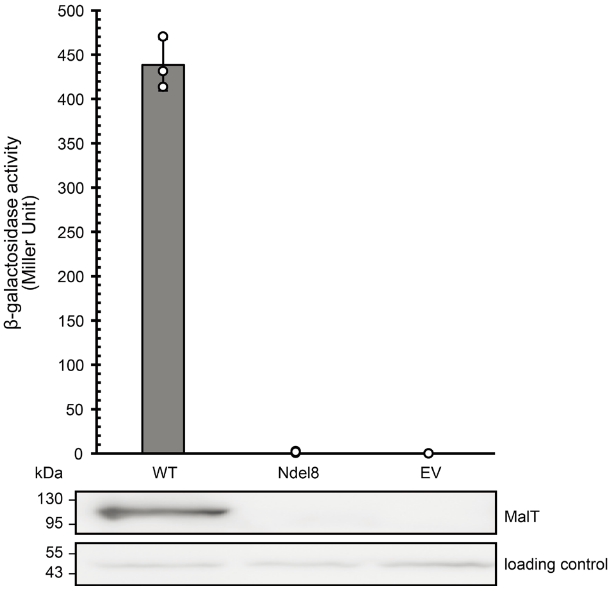

Supplementary Fig. 6 *In vivo* assay of MalT WT and mutant carrying a deletion of the N-terminal 8 amino acids (Ndel8). Levels of β-galactosidase activity in strains H harboring WT MalT plasmid (pJB215) or the derivative encoding Ndel8 and grown in a minimal medium supplemented with glycerol. The enzymatic activity values obtained were corrected for the background as measured with strains harboring empty vector (pJM241). The values given are the means ± SD of results from three independent experiments. MalT proteins were detected by western blot using total-cell extracts from the assayed cultures. A nonspecific band with lower molecular weight that appeared in all the samples was used as loading control.

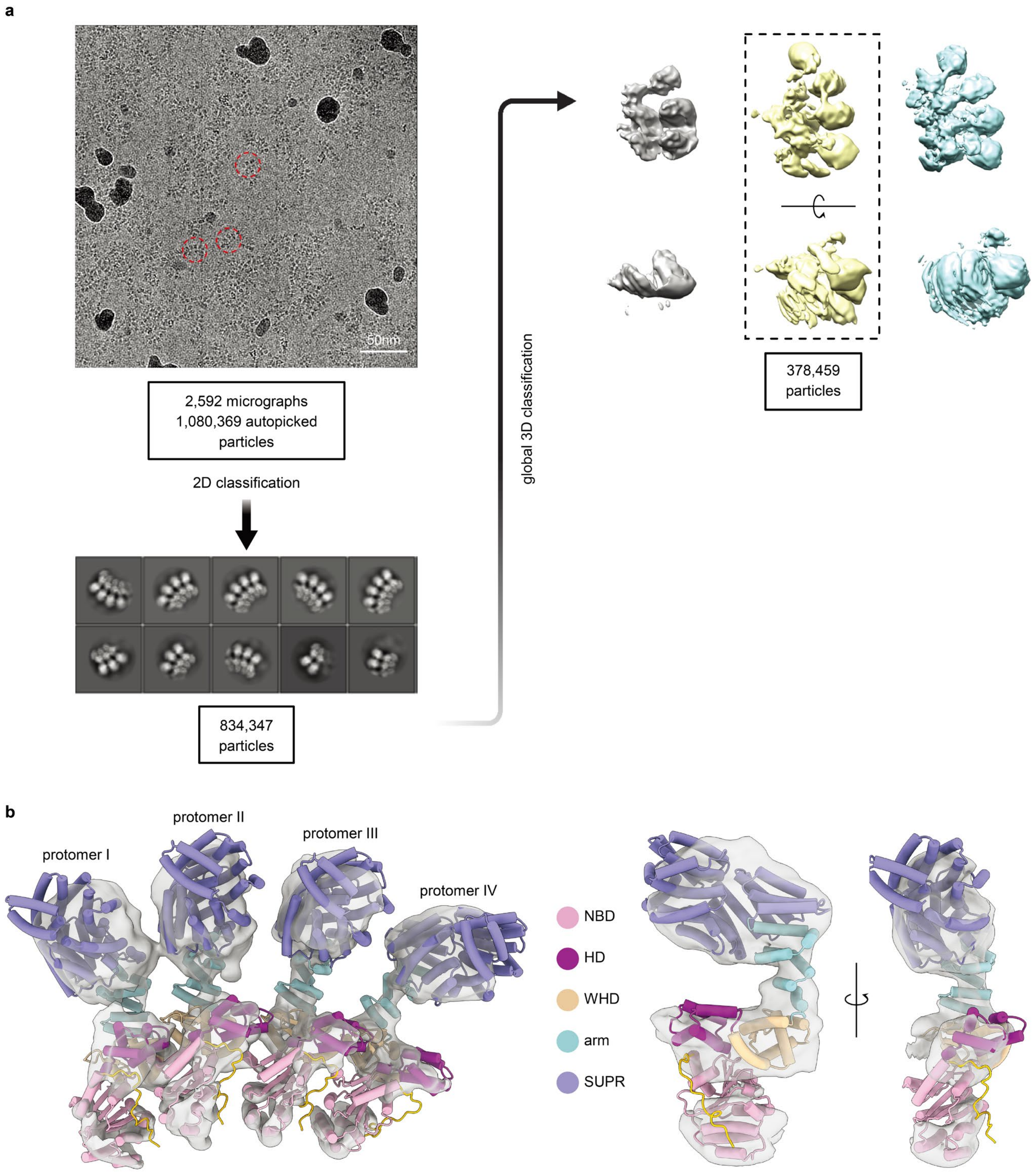

Supplementary Fig. 7 Cryo-EM workflow for oligomeric MalT. a, Flowchart for cryo-EM data processing and 3D reconstruction of MalT oligomer. **b**, Docking of the active MalT model predicted by Alphafold2[1] into the reconstructed 3D map. Colors of each protein domain are indicated, the N-terminal segment of NBD is highlighted in yellow.

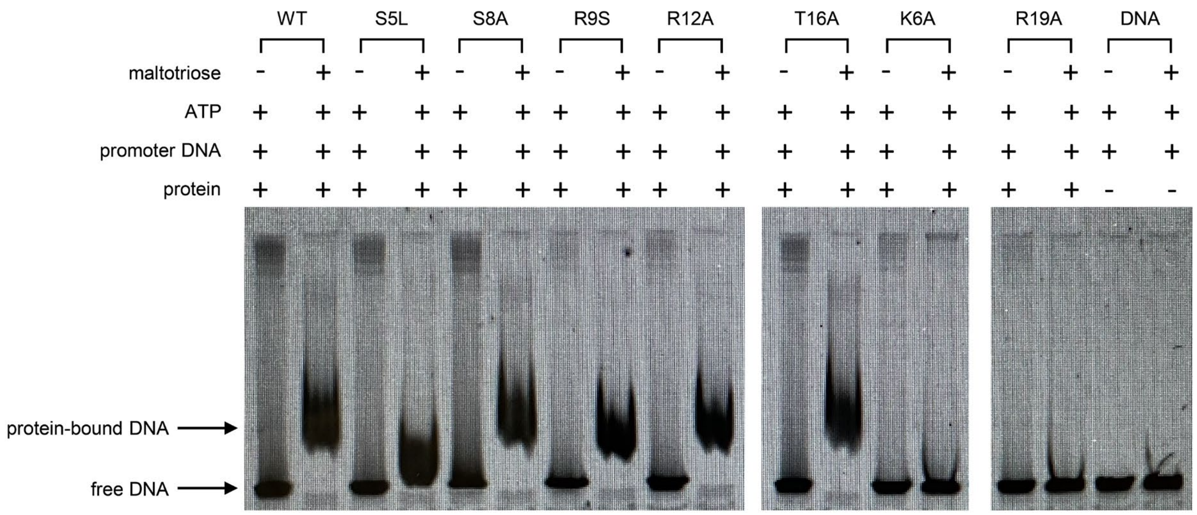

Supplementary Fig. 8 Impact of MalT N-loop mutations on MalT promoter-binding. 5 µM MalT WT or mutant proteins were incubated with 1 µM Cy3-labelled promoter *malPp800* fragment in the presence of 0.4 mM ATP, either with or without 1 mM maltotriose. Samples were run on native PAGE gels and DNA was detected with Cy3-specific filter. The experiments have been repeated for three times with similar results.

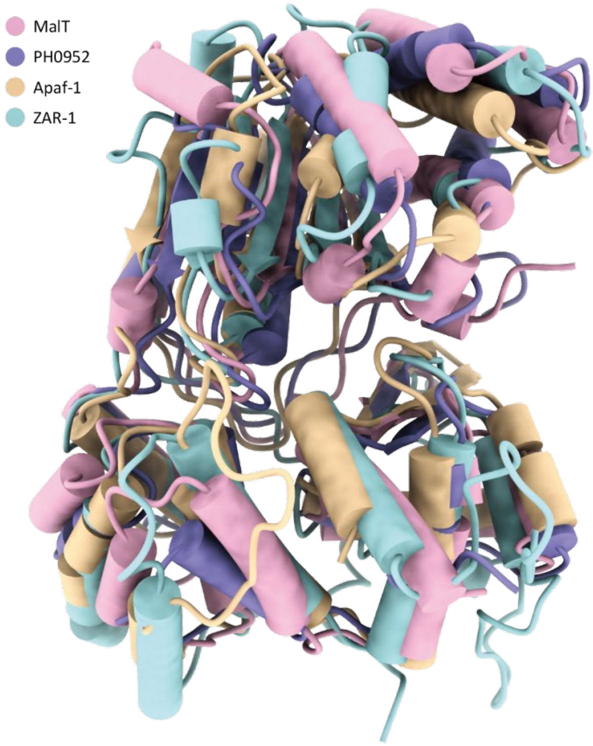

Supplementary Fig. 9 Structural alignment based on the NOD module. The NOD module of MalT (NBD, HD and WHD) was aligned to that from inactive PH0952 (slate), Apaf-1 (wheat) and ZAR-1 (cyan).

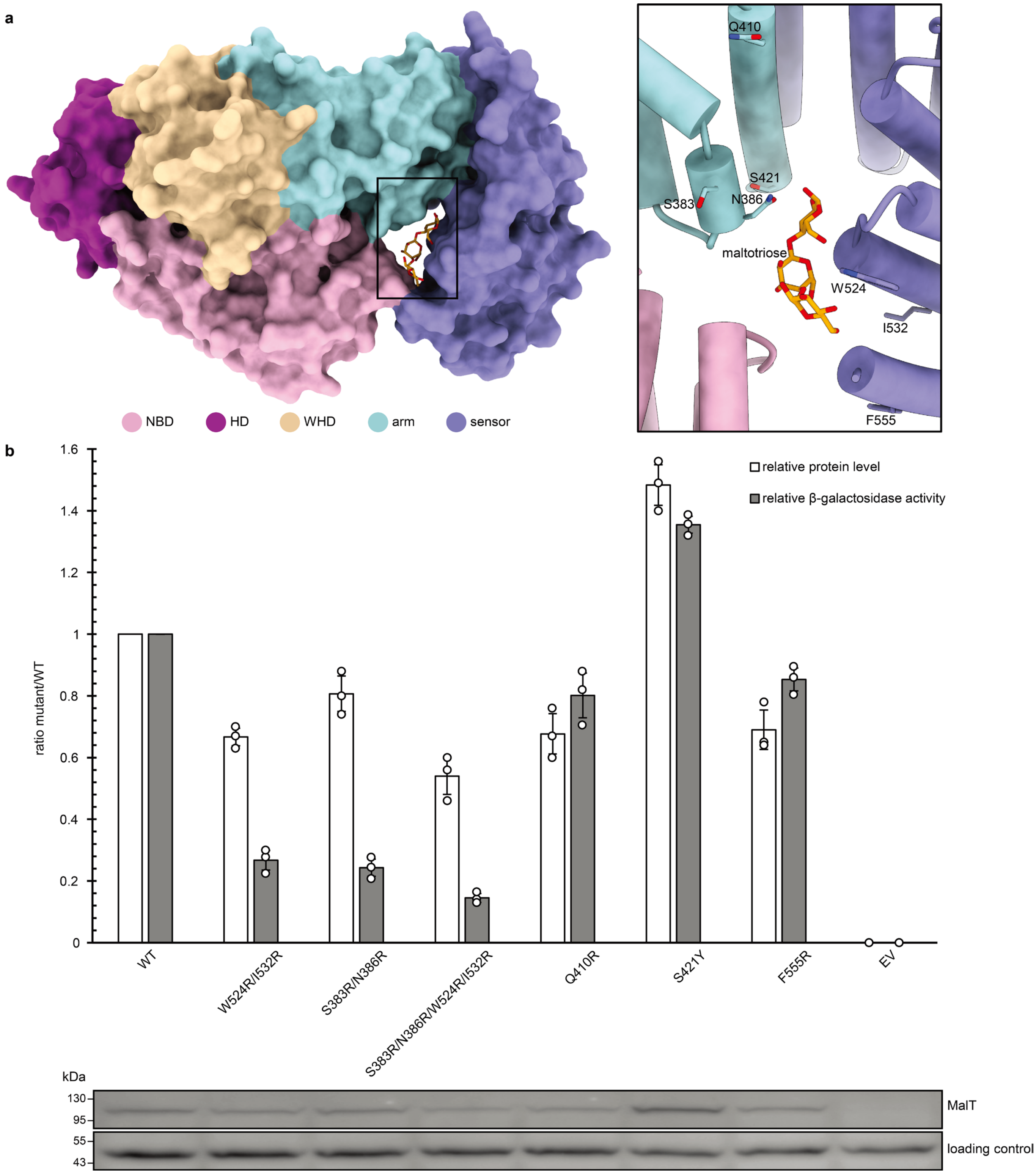

Supplementary Fig. 10 Mapping of maltotriose-binding site. a, Structure of inactive MalT without the DBD as predicted by AlphaFold2[1]. The black rectangle denotes the groove containing the maltotriose-binding site. Residues near the deduced maltotriose-binding site are shown. **b**, *In vivo* assay of MalT mutants carrying mutations near the deduced maltotriose-binding site. Residues that are potentially involved in maltotriose binding were identified on the predicted structure and substituted with bulky ones. The mutant protein activities were determined by measuring the levels of β-galactosidase in strain H harboring pJB215 or a derivative thereof and corrected for the background as described in Fig. 2, and the ratios of the mutant activities to that of WT were calculated. The relative protein levels of MalT mutant to that of WT were determined by western blot quantification using total-cell extracts from the assayed cultures. A non-specific band with lower molecular weight that appeared in all the samples was used as loading control. The values are the means ± SD of results from three independent experiments.

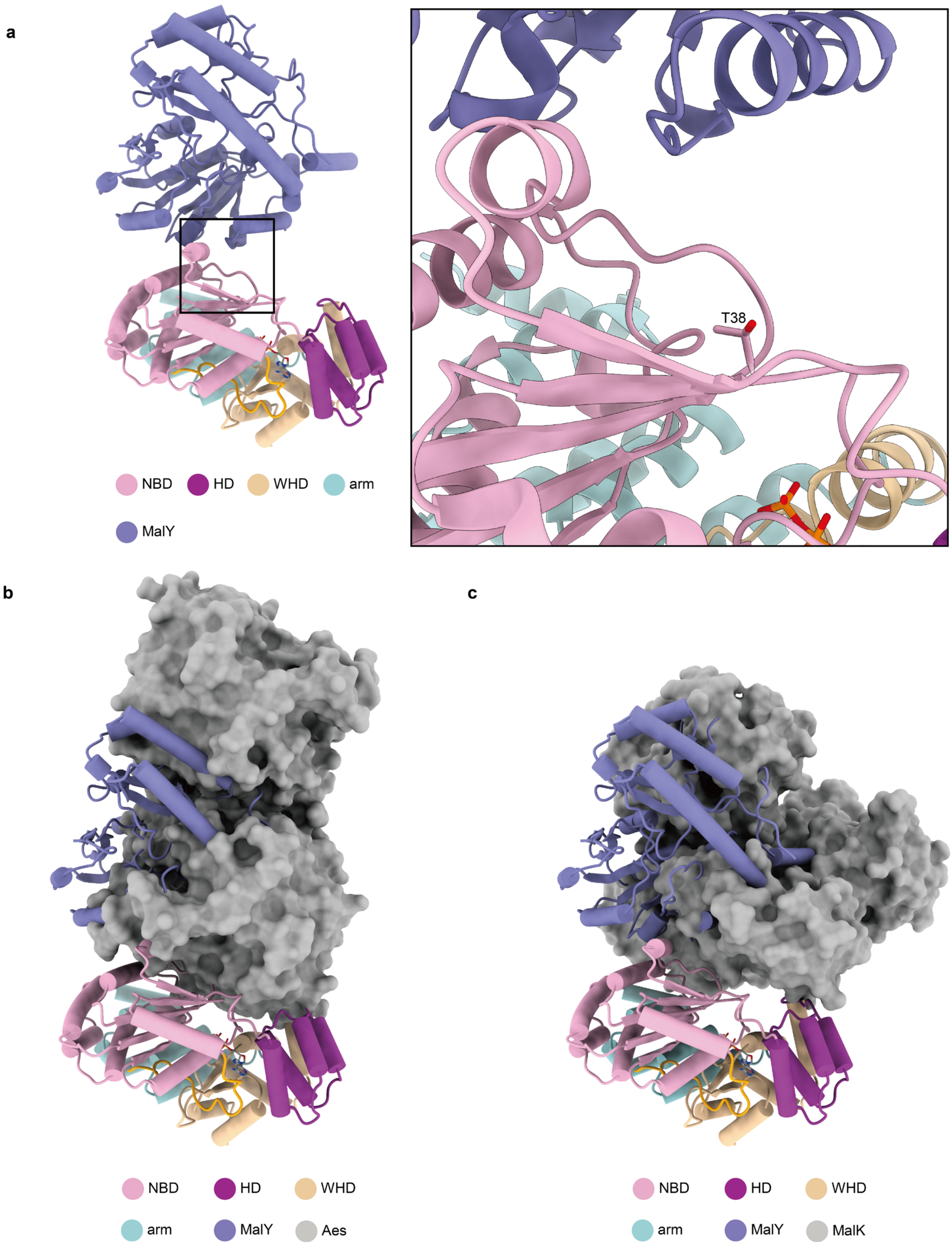

Supplementary Fig. 11 Interaction of MalT with MalY, MalK, and Aes. a, Position of residue T38 from MalT in the MalT-MalY complex. Structure of MalT-MalY is manually juxtaposed with **b**, an Aes dimer (PDB: 4KRX) or **c**, a MalK dimer (PDB: 3FH6). Residues reported to be involved in MalT regulation on MalT (T38) and Aes (R49, D122, D151) or MalK (W267, D297) are positioned facing each other[2-5]. Aes and MalK are shown in surface view. Colors of different domains and proteins are indicated, the N-terminal segment of NBD is highlighted in yellow.

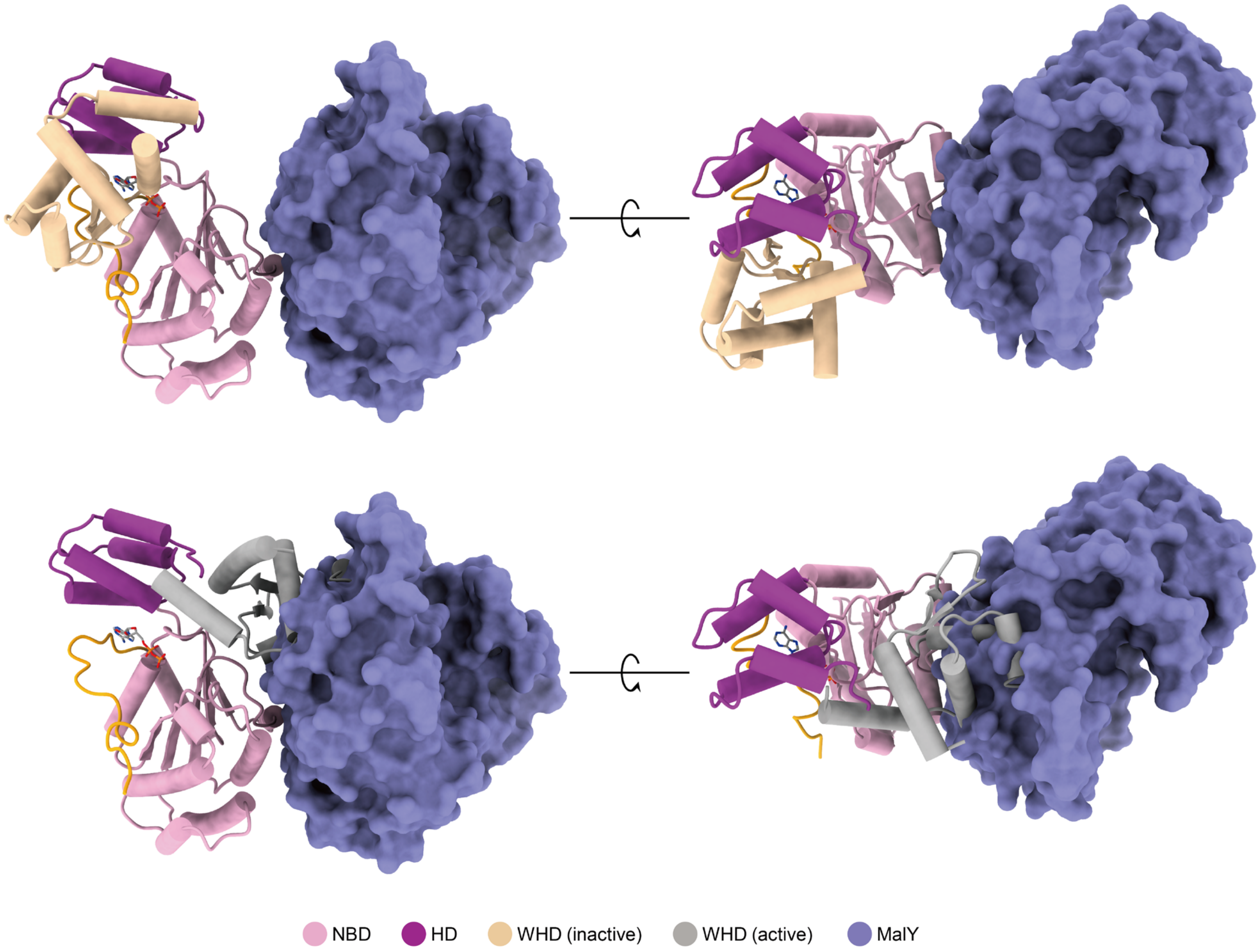

Supplementary Fig. 12 Structural remodeling of the NOD module during MalT activation. The WHD is distant from MalY in the MalT-MalY complex (top). An active MalT NOD module was modelled based on the active Apaf-1 NOD, in which the repositioning of WHD is incompatible with MalY from the MalT-MalY complex (bottom). Colors of different domains and proteins are indicated, the N-terminal segment of NBD is highlighted in yellow.

Supplementary Table 1 | Cryo-EM data collection, refinement and validation statistics

	MalT-MalY
PDB and EMDB ID	8BOB and EMD-16140
**Data collection**	
Cryo electron microscope	FEI Titan Krios
Voltage (kV)	300
Detector	Gatan K2 Summit
Energy filter slit width (eV)	20
Magnification	64,000×
Pixel size (Å)	1.061
Total electron exposure ($e^-/Å^2$)	50
Number of frames collected	32
Defocus range (μm)	-1.5 ~ -2.0
Automation software	AutoEMation
Micrographs collected	2,839
Micrographs used	2,802
**3D reconstruction**	
Software	RELION 3.1
Total extraced particles	1,145,822
Total number particles for final refinement	176,969
Symmetry imposed	C2
Resolution range (Å)	2.6~8.4
Resolution (Å) after refinement (FSC=0.143)	3.41
Resolution (Å) after post-processing (FSC=0.143)	2.94
Map sharpening B-factor ($Å^2$)	-60
**Refinement and validation**	
Software	Phenix.real_space_refine
Model resolution (Å)	3.0 (FSC=0.5)
Model composition	
Non-hydrogen atoms	12730
Protein residues	1606
B factors ($Å^2$)	214
R.M.S deviations	
Bonds lengths (Å)	0.005
Bonds angles (°)	0.649
MolProbity score	1.70
Clash score	7.26
Rotamer outliers (%)	1.43
Cb outliers (%)	0.00
CaBLAM outliers (%)	2.14
EMRinger score	3.48
Ramachandran plot statistics	
Preferred (%)	96.93
Allowed (%)	2.94
Outlier (%)	0.13

Supplementary References

1 Jumper, J. *et al.* Highly accurate protein structure prediction with AlphaFold. *Nature* **596**, 583-589 (2021). https://doi.org:10.1038/s41586-021-03819-2

2 Schlegel, A., Danot, O., Richet, E., Ferenci, T. & Boos, W. The N terminus of the Escherichia coli transcription activator MalT is the domain of interaction with MalY. *J Bacteriol* **184**, 3069-3077 (2002). https://doi.org:10.1128/JB.184.11.3069-3077.2002

3 Khare, D., Oldham, M. L., Orelle, C., Davidson, A. L. & Chen, J. Alternating access in maltose transporter mediated by rigid-body rotations. *Mol Cell* **33**, 528-536 (2009). https://doi.org:10.1016/j.molcel.2009.01.035

4 Schiefner, A., Gerber, K., Brosig, A. & Boos, W. Structural and mutational analyses of Aes, an inhibitor of MalT in Escherichia coli. *Proteins* **82**, 268-277 (2014). https://doi.org:10.1002/prot.24383

5 Lisa, M. N. *et al.* Double autoinhibition mechanism of signal transduction ATPases with numerous domains (STAND) with a tetratricopeptide repeat sensor. *Nucleic Acids Res* **47**, 3795-3810 (2019). https://doi.org:10.1093/nar/gkz112
