# Supplementary material for: Structural basis for negative regulation of the Escherichia coli maltose system
Source: Nat Commun. 2023 Aug 15;14:4925. doi: 10.1038/s41467-023-40447-y (PMC10427625; doi:10.1038/s41467-023-40447-y)
Supplement: Supplementary file 3 — Reporting Summary [file 41467_2023_40447_MOESM3_ESM.pdf]

nature portfolio

Corresponding author(s): Jijie Chai

Last updated by author(s): Jul 7, 2023

Reporting Summary

Nature Portfolio wishes to improve the reproducibility of the work that we publish. This form provides structure for consistency and transparency in reporting. For further information on Nature Portfolio policies, see our Editorial Policies and the Editorial Policy Checklist.

Statistics

For all statistical analyses, confirm that the following items are present in the figure legend, table legend, main text, or Methods section.

n/a	Confirmed	
☐	☒	The exact sample size (*n*) for each experimental group/condition, given as a discrete number and unit of measurement
☐	☒	A statement on whether measurements were taken from distinct samples or whether the same sample was measured repeatedly
☐	☒	The statistical test(s) used AND whether they are one- or two-sided<br>*Only common tests should be described solely by name; describe more complex techniques in the Methods section.*
☒	☐	A description of all covariates tested
☐	☒	A description of any assumptions or corrections, such as tests of normality and adjustment for multiple comparisons
☐	☒	A full description of the statistical parameters including central tendency (e.g. means) or other basic estimates (e.g. regression coefficient) AND variation (e.g. standard deviation) or associated estimates of uncertainty (e.g. confidence intervals)
☐	☒	For null hypothesis testing, the test statistic (e.g. *F*, *t*, *r*) with confidence intervals, effect sizes, degrees of freedom and *P* value noted<br>*Give P values as exact values whenever suitable.*
☒	☐	For Bayesian analysis, information on the choice of priors and Markov chain Monte Carlo settings
☒	☐	For hierarchical and complex designs, identification of the appropriate level for tests and full reporting of outcomes
☒	☐	Estimates of effect sizes (e.g. Cohen's *d*, Pearson's *r*), indicating how they were calculated

Our web collection on statistics for biologists contains articles on many of the points above.

Software and code

Policy information about availability of computer code

Data collection	Blu-Ice BL19U1, AutoEmation2.
Data analysis	RELION 3.1, MotionCor2 1.0.5, CTFFIND 4.1.12, Phenix 1.18, Chimera 1.14, Coot 0.8.9, ChimeraX 1.5, MolProbity 4.1.

For manuscripts utilizing custom algorithms or software that are central to the research but not yet described in published literature, software must be made available to editors and reviewers. We strongly encourage code deposition in a community repository (e.g. GitHub). See the Nature Portfolio guidelines for submitting code & software for further information.

Data

Policy information about availability of data

All manuscripts must include a data availability statement. This statement should provide the following information, where applicable:

- Accession codes, unique identifiers, or web links for publicly available datasets
- A description of any restrictions on data availability
- For clinical datasets or third party data, please ensure that the statement adheres to our policy

Source data are provided with this paper. All the data needed to evaluate the conclusions of the manuscript are present in the paper or the Supplementary Information. The cryo-EM density maps and corresponding atomic coordinates for the MalT-MalY complex have been deposited in the Electron Microscopy Data Bank (EMDB) and Protein Data Bank (PDB) under accession number EMD-16140 and 8BOB. Other PDB entries mentioned in this study include 6MFV, 1D2F, 4KRS, and 3FH6. All other data are available from the corresponding author on request.

Human research participants

Policy information about studies involving human research participants and Sex and Gender in Research.

Reporting on sex and gender	N/A
Population characteristics	N/A
Recruitment	N/A
Ethics oversight	N/A

Note that full information on the approval of the study protocol must also be provided in the manuscript.

Field-specific reporting

Please select the one below that is the best fit for your research. If you are not sure, read the appropriate sections before making your selection.

- [x] Life sciences
- [ ] Behavioural & social sciences
- [ ] Ecological, evolutionary & environmental sciences

For a reference copy of the document with all sections, see nature.com/documents/nr-reporting-summary-flat.pdf

Life sciences study design

All studies must disclose on these points even when the disclosure is negative.

Sample size	For β-galactosidase assays, three independent cultures were used for measuring the enzymatic activity of each variant. For western blot quantification, band intensity was measured three times from three individual blots. The amount of protein-bound nucleotides was quantified from three independent samples of each variant by luciferase assays. Three biological replicates were sufficient for the observations and claims made in this study.
Data exclusions	Data exclusion for particles in bad 2D or 3D classes with no defined features was a standard processing for EM data.
Replication	To ensure reproducibility of experimental findings, each assay was performed at least three times to confirm the results, with all attempts at replication successful.
Randomization	Randomization was not necessary. All independent variables and associated observations were sufficient for the interpretation and claims made within this study.
Blinding	Blinding was also not necessary for experimental data acquired in this study, and all the data are subject to statistical analyses when necessary.

Reporting for specific materials, systems and methods

We require information from authors about some types of materials, experimental systems and methods used in many studies. Here, indicate whether each material, system or method listed is relevant to your study. If you are not sure if a list item applies to your research, read the appropriate section before selecting a response.

Materials & experimental systems

n/a	Involved in the study	
[ ]	[x]	Antibodies
[x]	[ ]	Eukaryotic cell lines
[x]	[ ]	Palaeontology and archaeology
[x]	[ ]	Animals and other organisms
[x]	[ ]	Clinical data
[x]	[ ]	Dual use research of concern

Methods

n/a	Involved in the study	
[x]	[ ]	ChIP-seq
[x]	[ ]	Flow cytometry
[x]	[ ]	MRI-based neuroimaging

Antibodies

Antibodies used	Peroxidase-conjugated anti-HA antibody (Roche, clone 3F10, 12013819001) used at 50 mU/mL.
Validation	The Roche 3F10 antibody used here is a frequently used antibody across a multitude of papers, and was internally validated against

empty vector loading controls in all western blots.
